# Is there an intergenerational discrepancy in the comprehension and aesthetic preference regarding emoji usage? Evidence from WeChat

**DOI:** 10.3389/fpsyg.2024.1424728

**Published:** 2024-07-11

**Authors:** Donghang Wu, Xinxiu Zhang, Xinjia Zhang

**Affiliations:** ^1^School of Fine Arts, Northeast Normal University, Changchun, China; ^2^School of Design and Art, Jingdezhen Ceramic University, Jingdezhen, China; ^3^School of Arts and Design, Yanshan University, Qinhuangdao, China

**Keywords:** generational interaction, emojis, comprehension biases, aesthetic preferences, WeChat

## Abstract

Emojis are widely used on social media, blogs, and instant messaging to express users’ feelings. However, in everyday interactions, the same emoji often has different interpretations and aesthetic preferences among different age groups. This can lead to communication barriers and misunderstandings. Based on social identity theory, this study uses WeChat, a social platform popular in China, to analyze intergenerational differences in emoji understanding and preferences through a questionnaire survey. The results indicate: (1) There are significant intergenerational differences in the usage habits, interpretation, and aesthetic preferences of emojis. (2) Middle-aged and elderly tend to interpret goodbye emoji symbols as simple emotional expressions, such as “goodbye” or “see you later,” while younger-age groups lean towards more complex emotions and social intentions, such as “speechlessness” and “end of friendship.” (3) Younger-age groups use emojis frequently and with a wide variety, whereas middle-aged and elderly groups use emojis less frequently and with limited variety. Younger individuals’ aesthetic preferences for emojis lean towards humor, conflict, and narrative, whereas middle-aged and elderly groups prefer emojis with bright colors and everyday greetings typical of their generation. Based on research findings, we believe that social identity theory provides a framework for understanding how individuals establish their identities through interactions with specific social groups. This study is beneficial for identifying the comprehension and aesthetic biases in emoji usage across generations, sheds light on the broader implications of social identity theory in digital communication contexts, and promotes friendly social interactions in real-time communication applications.

## 1 Introduction

The advent of smartphones as handheld computers has heralded the era of mobile internet, facilitating the transcending of temporal, spatial, and geographic barriers. This empowerment allows individuals to participate in online communication, social networking, and information sharing at their convenience and from any location (Hancock et al., 2024). As a result, there has been a proliferation of chat applications, such as WeChat, Mobile Fetion, UCTalk, and Yahoo Messenger, alongside social networking platforms like TanTan, Momo and Soul. Emojis with their intuitive, convenient, and emotionally expressive features, continue to captivate people’s visual senses, becoming pivotal vehicles for social networking and information exchange. Emojis are not merely extensions of visual images but also the fusion of emotions and connotations. They have revolutionized the way people communicate, making interactions between individuals more vivid and diverse, greatly facilitating the exchange of information and emotions (Was and Hamrick, 2021). Emojis are pictographic symbols used in web pages and chats. They originated as visual emotional symbols in Japanese wireless communication and are particularly popular in Asia (Bai et al., 2019; Franco and Fugate, 2020). During the late 20th century, novel terms emerged in English to delineate these emotive symbols. Specifically, the fusion of “Emotion” and “Icon” coined the neologism “Emoticon” (Urabe et al., 2021). Subsequently, this non-verbal mode of expression gained prominence, transitioning from text to images, from static to dynamic forms, and leveraging varied combinations of text, symbols, and graphics to emulate emotions, postures, and actions. Remarkably, it mirrors individuals’ real-life emotions, situations, and expressions in the realm of social media dissemination with remarkable speed. According to Unicode statistics, 92% of global internet users also used emojis in 2021 (Daniel and Camp, 2020).

According to the 7th National Population Census of China (2021), the proportion of individuals aged 60 and above has reached nearly 19%, marking a 2.6% increase from 5 years ago. The maturation and advancement of mobile terminal technology in internet communication have led to the elderly becoming a significant consumer group of mobile devices. The acceleration of China’s aging population, coupled with the proliferation of smart technology and consumerism, has spurred middle-aged and elderly individuals to actively participate in online social activities. The “2018 WeChat Annual Report” provided insights into emoji usage preferences across age demographics. It highlighted that individuals born in the 1970s, 1980s, 1990s, and 2000s display diverse preferences and frequencies in emoji usage. From the perspective of cultural metaphors, Pan (2023) observes that the younger generation is the primary creators, users, disseminators, and names of emoji packs featuring middle-aged and elderly individuals. By employing metaphors, youth imbue these emoji packs with multiple meanings from the outset, effectively turning them into a form of “code” during social interactions. This facilitates specialized communication and strengthens group identity. In everyday communication, the elderly utilize emojis to engage with family and friends, thereby enhancing the liveliness and interest of conversations while facilitating the intuitive understanding and clarification of written language (Hu, 2017; Cui et al., 2024). Nonetheless, disparities in generational ideologies, perspectives, and values contribute (Luescher and Pillemer, 1998) to the emergence of “generation gap or social identity theory” issues in emoji-based interactions.

Previous scholarly investigations have explored emojis across various academic disciplines, including psychology (Upadhyay et al., 2023), semiotics, communication studies, and sociology (Logi and Zappavigna, 2023). Research methods include surveys, interviews, online surveys, controlled experiments, and artificial neural networks (Robus et al., 2020; Paggio and Tse, 2022; Yang et al., 2023). Analytical methods employed comprise emotion arousal and valence assessment, semantic modeling, Pearson correlation analysis, regression analysis, and variance analysis (Cappallo et al., 2019; Cherbonnier and Michinov, 2021; Shardlow et al., 2022; Kaye et al., 2023; Kaye and Schweiger, 2023). These studies have examined the semantic interpretations and emotional connotations associated with emojis across different countries, digital platforms, and social media platforms (such as Apple, Android, Samsung, Twitter, and WeChat), as well as within different demographic groups based on gender and age (Fischer and Herbert, 2021; Wei, 2021). Cultural disparities exist across generations regarding the utilization of emojis to convey sarcasm or ironic undertones. Moreover, interpretations of emojis vary between younger and older individuals. Hsiao and Hsieh (2014) analyzed older adults to have a more positive response toward perceived emojis than younger ones. The two age ranges have different cognition of the design appearances of realistic and abstract emojis. An et al. (2018) found people between 26 and 35 had the lowest frequency of emoji usage. Younger and elder groups showed different sentiment levels for the same emojis. People chose emoji types based on relationships. Weiß et al. (2020) found that as age increases, the positivity of smiles also rises. Older individuals exhibit more pleasure and positive emotions towards emojis. Herring and Dainas (2020) suggest that respondents aged 30 and above tend to interpret emojis literally. Among older males, there is a higher likelihood of misunderstanding the function of emojis, whereas younger females are least likely to misunderstand the function of emojis. Cui (2022) conducted two experiments to examine differences in the usage of smiling emojis between young and elderly individuals. For young participants, the sender’s age and relationship with the recipient were significantly correlated with the ironic interpretation of emojis. However, for older individuals, the sender’s age did not affect on the ironic interpretation of emojis. Gallud et al. (2018) reveal no significant difference between older adults and younger age groups in the use of emojis, although older adults experience more difficulty in interpreting them. Wu (2016) analyzed the value and design styles of emojis. Yu (2018) explored the aesthetic intentions behind emojis by examining their design genesis, evolution, and stages of popularity. Prada et al. (2018) discovered that both young individuals and females employ emojis more often in their everyday digital interactions. Additionally, younger participants expressed more compelling reasons for utilizing emojis, such as aiding in emotional expression, reinforcing message content, and even mitigating the tone of the message.

## 2 Theoretical foundations: social identity theory

Social Identity Theory (SIT), proposed by Tajfel and Turner (1979), posits that individuals derive a portion of their self-concept from their membership in social groups. The theory seeks to explain the cognitive processes and social conditions underlying intergroup behaviors, especially those related to prejudice, bias, and discrimination (Wang et al., 2024a,b). SIT encompasses multiple sub-theories. One sub theory is intergroup similarity, which posits that similar groups have a greater motivation to distinguish themselves from each other (Brown, 2000; Harwood et al., 2005). The group similarity theory suggests that similar groups have higher intergroup attractiveness and lower intragroup bias. Social identity is conceptualized as one’s membership in social groups that have emotional and value significance attached to them. Individuals who identify themselves as members of a group are expected to seek positive distinctiveness for the in-group compared to the out-group. Positive distinctiveness is achieved through in-group favoritism and out-group denunciation, which can include cognition and behaviors driven by one’s social identity (Tajfel and Turner, 1986).

Individuals often compare their group’s social status or identity with other groups to maintain or achieve a positive distinctiveness. This can lead to self-enhancement or increased self-esteem (Rubin and Hewstone, 1998). Social categorization refers to the tendency of people to classify themselves and others into various social groups based on attributes like age, race, gender, nationality, or religion. Categorization helps individuals simplify the social environment. Once individuals categorize themselves as members of a particular group, they adopt the identity of that group. This means they begin to see themselves in terms of group characteristics and adopt its norms, values, and behaviors. After categorizing and identifying with a group, individuals compare their group to others. This comparison is often biased in favor of one’s own group, leading to in-group favoritism. The “in-group” refers to the group with which an individual identifies, while “out-group” pertains to groups they do not identify with. The theory asserts that people have a natural inclination to perceive their in-group in a positive light while being neutral or even negative towards out-groups, thus enhancing their self-image. For age social groups, such as Teenagers as an inner group, might feel that other teens understand their experiences and challenges best, they might see adults, especially older adults, as an outgroup (Tajfel and Turner, 2004).

Taking age as a dimension of identity, the categorization labels most frequently acknowledged by both researchers and respondents in intergenerational differences are young, middle-aged, and elderly (Harwood et al., 1995). Chinese society categorizes each generation by decade, such as post-2000s, post-90s, post-80s, post-70s, and post-60s. The most direct manifestation of these demographic changes is generational differences (Liao, 2004). The “2023 China Generational Insights Report” by Massive Arithmetic (China’s massive computing platform is a big data analysis tool under ByteDance) and New Weekly identified 20 typical generational groups since the founding of New China (After 1949). Using TikTok ecosystem big data, the report extracted their basic profiles, content preferences, and decision-making psychology. Chinese generational groups exhibit four distinct characteristics: (1) Different generational groups are relatively conservative in consumption and have a clear demand for saving. (2) A turning point in the shift from valuing tradition to valuing self first emerged between the post-70s, post-80s, and post-90s. (3) Younger generations are more diverse in their views on the same issues showing a trend of diversification. (4) Younger generations favor search engines, with this preference becoming more pronounced in younger generations. These findings demonstrate the differences among generational groups in terms of culture, social groups division, and attitudes toward electronic information and big data. For example, Lin et al. (2017) analyzed that the generational digital divide between youth and other age groups is mainly studied in terms of differences in WeChat usage and liking behaviors (Chen and Shao, 2018). Zhao (2019) found that the post-70s generation is generally weaker than the post-80s and post-90s generations in terms of internet information acquisition, social interaction, public participation, self-interaction, leisure and entertainment, online learning, and life assistant use. This generational difference stems from different points of internet access and life circumstances representing the third digital divide. Pan (2023) discussed that the post-2000s generation places more emphasis on achievement motivation and the value of self-actualization, which is reflected in a new form of cultural participation.

Social identity theory frequently utilizes qualitative research (Pandey et al., 2014) and thematic analysis of interview data. This method has been effectively applied to studies of workplace social identity (Wang et al., 2022) and gay identity (Hajek, 2014). Emojis, as a form of expression in the digital age, can reflect this form of identity expression and communication in social media and online communication. Emoji is not only a tool for expressing emotions and tone, but also a symbolic expression of identity and group identity. Völkel et al. (2019) showed that the usage of some emoji is correlated with aspects of identity such as personality. Li et al. (2020) confirmed that homophily effects exist concerning the types of emoji that are included in the bios of users and their followers. Gervasio and Karuri (2019) found that convergence is realized through students’ resemblance in their language usage on social media. These studies indicate that similarities or differences between individuals or groups can influence emoji use and interpretation. While existing research employs SIT to explain generational differences in social media use, the specific role of emojis as social symbols remains unclear. Additionally, there is a lack of comparative analysis of SIT across different generational age groups.

Existing research extensively examines the semantic and emotional understanding of sarcasm in emoji use among young and elderly individuals (Jaeger et al., 2018; Kutsuzawa et al., 2022; Boutet et al., 2024). The majority of empirical studies have centered on English-speaking populations (Neel et al., 2023), neglecting to investigate the linguistic and cultural contexts of the Chinese language. Additionally, the comparative mechanisms of intergenerational differences in emoji use and aesthetic preferences based on social identity theory are largely unexplored. As shown in the above literature, individuals from various age cohorts exhibit divergent tendencies in aesthetic selection and understanding of emojis. Therefore, there is a need to pay attention to comprehending the discrepancies in emoji usage across different generations.

This study examines emojis used in WeChat among Chinese-speaking young and elderly individuals. Using social identity theory, it investigates generational biases in emoji interpretation through a questionnaire survey. The study analyzes visual aesthetic preferences for emojis across generations and explains why different age groups may have varying preferences and interpretations based on SIT. Theoretical and practical implications are discussed. Theoretically, this enriches research on the comparative mechanisms of SIT regarding intergenerational differences. It enhances our understanding of how digital communication tools reflect and shape group identities across different age groups. Practically, this study seeks to enhance emoji design, recommendation algorithms, and street marketing strategies on social media platforms (Rong et al., 2022). These enhancements boost internal communication efficiency within companies and foster friendly, sustainable networking on real-time communication apps for developers, marketers, and general users.

Its research hypotheses are as follows:

1. There are significant differences among different age groups in the comprehension and aesthetic aspects of emojis.
2. Social contexts, cultural differences, generational identities, and social cognition are key factors that influence intergenerational choice of emojis.

## 3 Research methods and data

### 3.1 Participants

WeChat is the most widely used instant messaging app in China. As of 2022, it has 1.31 billion monthly active users. Of these, 36% are under 30 years old, 41.3% are between 31 and 50 years old, and 22.7% are over 50 years old, demonstrating a broad age demographic (Shubham, 2024). This study aligns the age classifications with those defined by the United Nations World Health Organization to match the age demographics of WeChat users. Age categorization in the questionnaire adhered to the World Health Organization’s guidelines: 0–17 for minors (after 2006) (data not collected due to minimal mobile phone usage), 18–44 for young adults (2005–1979), 45–59 for Middle-aged groups (1978–1964), and 60 years and above for elderly (before 1963).

This study utilized an online survey format for data collection due to its benefits, including large sample size, low cost, high time efficiency, wide geographical coverage, and diverse question types. The data collection procedures have been approved by the Institutional Review Board of Northeast Normal University. We surveyed emoji usage in the popular social media app WeChat using the online platform WJX,^1^ a crowdsourcing platform in mainland China. The survey took place from November 1st to November 30th, 2023. Participants are recruited online through opportunistic sampling. They access the survey instructions by clicking on the survey webpage link. Participants voluntarily respond to research questions and can withdraw from the survey at any time. The questionnaire data were utilized solely for research purposes and treated as confidential. Hence, all participants provided informed consent and participated voluntarily. The survey collected 376 questionnaires, of which 369 were valid (some samples were incomplete). Participants included 135 individuals aged 18–44, 128 middle-aged individuals aged 45–59, and 106 elderly individuals aged 60 and above. The similar number of participants across these age groups ensures balanced and reliable data analysis, as indicated in Table 1.

**Table 1:** Basic information of the questionnaire.

Category	Options	Frequency	Percentage	Cumulative percentage
Age	17–44	135	36.6	36.6
	45–59	128	34.7	71.3
	60+	106	28.7	100.0
Total		369	100.0	100.0

### 3.2 Emoji classification

Emojis are a form of ideograms, consisting of icons intended to represent facial expressions, emotions, objects, or other symbols, most commonly used in technologies such as smartphones, tablets, and computers (Was and Hamrick, 2021). With the advancement of online information, emojis have further evolved in terms of expression and themes. The types of presentation include emoticons, emojis, image-based emojis, and memes. Thematic categories include film and television, celebrity and internet celebrity, cartoon and animation, flora and fauna, and natural scenery, as shown in Table 2. The emojis examined in this study are based on the most popular ones in China from 2020 to 2023 and those that have garnered scholarly attention (Du, 2020; Han, 2022).

**Table 2:** Classification of emoticons.

Emoji	Types	Content	Diagram	Diagram
Presentation type	Emoticons	ASCII, text emoji, composed of combinations of punctuation marks, Arabic numerals, and letters, with diverse combinations.	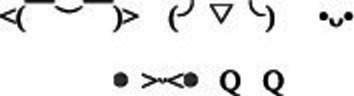	This series of emoticons is composed of ASCII characters and does not have a specific origin.
	Emoji	Emoji symbols feature simple and uniform styles, wide coverage, and easy, fast use. They are applied in various social media platforms’ built-in emoji systems.	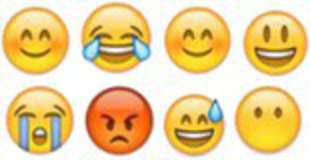	https://joypixels.com/emoji
	Image-based	Images can be static, dynamic, or a combination of both.	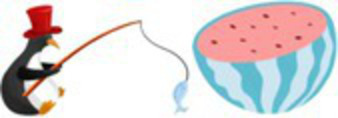	https://www.sohu.com/a/354466506_100293619 https://www.sohu.com/a/469884242_121124019
	Meme	An image or video that is spread widely on the internet, often altered by internet users for humorous effect.	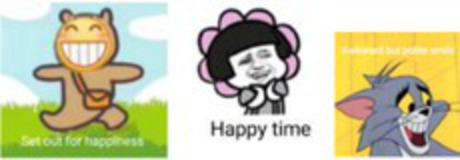	https://www.sohu.com/a/311278820_197613 https://www.sohu.com/a/353493350_99940893 https://www.sohu.com/a/582591181_121145572
Theme type	Film and television works	The materials mainly come from films, TV shows, and animated videos, mainly made by capturing frames, which can be static or dynamic images.	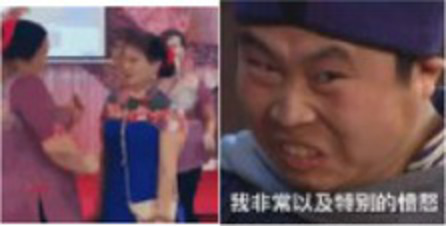	https://www.bilibili.com/video/BV1Tj411v7Ny/?spm_id_from=333.788.recommend_more_video.1 https://www.sohu.com/a/513311780_99966042
	Celebrity and internet celebrity	The materials mainly come from celebrities and internet celebrities, mainly in two types: ① directly captured or cut from photos or videos; ② cartoonized portraits.	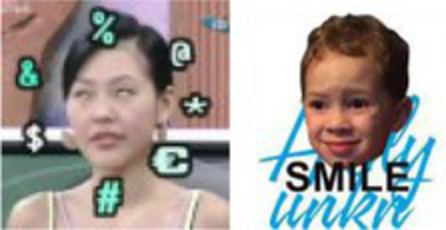	https://www.sohu.com/a/680643616_100192528 https://www.photophoto.cn/sucai/29958526.html
	Cartoon and animation	The materials mainly come from popular cartoons or self-created cartoon characters, which can be characters, animals and plants, or fictitious non-natural objects.	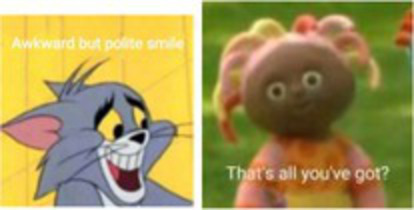	https://www.sohu.com/a/582591181_121145572 https://k.sina.cn/article_2801138254_a6f5fa4e04000x31z.html
	Flora and fauna	The materials mainly come from images of animals and plants, mostly cute pets and adorable plants.	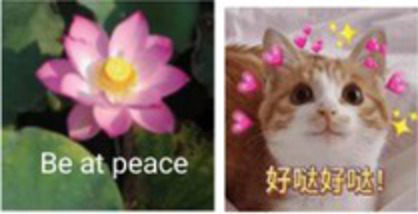	https://m.duitang.com/blogs/tag/?name=%E5%9C%9F%E5%91%B3%E5%A4%B4%E5%83%8F https://gxwmz.com/qianming/qqbiaoqing/m87pzk.html
	Natural landscapes	This category mainly includes natural landscapes, such as mountains, rivers, trees, flowers, and plants.	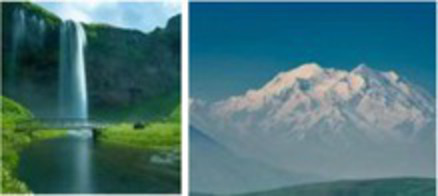	https://m.qulishi.com/article/201809/295326.html https://m.k366.com/sxnf/2022/159333.html

### 3.3 Procedure

Considering the unique and complex use of emojis across generations, the proposed questionnaire will be sent to 10 professors from Chinese universities and 10 digital media design peers for feedback and revision. The questionnaire includes single-choice and multiple-choice questions covering various aspects such as emoji usage, understanding biases, aesthetic preferences, and design preferences. Each question has been pre-tested and reviewed by experts to ensure validity and reliability. The emoji usage section will collect data on age, social media usage time, emoji usage habits, number of emojis saved, usage targets, contexts, and reasons for use. Understanding biases will consider participants’ interpretations of specific emojis and the reasons and challenges of differing interpretations across generations. Aesthetic preferences will focus on participants’ preferred emoji styles, types, themes, and preferences for emojis in specific emotional contexts. Design preferences will be measured through design methods, colors, and fonts. We apply social identity theory and Pearson’s Chi-square test (*χ*^2^). *χ*^2^ test will be conducted using SPSS software to explore differences in emoji understanding and usage across different age groups.

## 4 Results

### 4.1 Usage analysis of emoji symbols

#### 4.1.1 Emoji symbol usage frequency

According to statistical data (see Table 3), there are significant differences in social media usage time and habits among different age groups. For social media usage time, most individuals aged 60 and above spend 1–3 h daily on social media (66 respondents). In contrast, the 18–44 age group shows more diverse usage, with a significant number spending 3–5 h (41 respondents) and over 5 h (38 respondents). The 45–59 age group primarily spends 1–3 h (55 respondents), followed by 3–5 h (38 respondents). Regarding emoji usage habits, the 18–44 age group uses emojis most frequently, with 68 respondents using emojis often and 36 almost always using them. The 60 and above age group uses emojis less frequently, with 47 respondents using emojis often and none heavily relying on them. The 45–59 age group has a balanced usage of emojis. Regarding the number of saved emojis, individuals aged 18–44 tend to save more, with 35 respondents having over 100 emojis. Middle-aged groups generally save 20–80 emojis, while older participants mostly save fewer than 20 emojis.

**Table 3:** Analysis of intergenerational use of emojis.

	Options	Age			Total
		18–44	45–59	60+	
How much time do you spend on social media apps each day?	Less than 1 h	9	18	26	53
	1–3 h	47	55	66	168
	3–5 h	41	38	14	93
	Over 5 h	38	17	0	55
Do you have a habit of using emoji symbols?	Almost always, chat heavily relies on using emoji	36	9	0	45
	Frequently, communication relies heavily on emoji packs	68	38	47	153
	Occasionally, to supplement text content	31	81	59	171
How many emoji symbols do you have saved on your phone?	20–50	14	45	42	101
	50–80	32	45	27	104
	Less than 20	37	25	25	87
	80–100	17	6	11	34
	Over 100	35	7	1	43
Whom do you typically use emoji symbols for?	Family	62	50	73	185
	Friends	81	65	63	209
	Classmates	69	39	6	114
	Partner	56	29	25	110
	Teacher	23	11	1	35
	Colleagues	42	33	2	77
	Leader	15	9	5	29
	Anyone	23	17	20	60
In what context do you typically use emoji symbols?	Feeling relaxed and happy	69	83	63	215
	Feeling awkward and uncertain	42	32	22	96
	Feeling angry and upset	26	28	24	78
	Use in any situation	82	40	36	158
What are your reasons for using emoji symbols?	Convenient and efficient, reducing typing time	72	62	67	201
	Lively and engaging, fostering communication atmospheres	98	52	29	179
	Softening tones and avoiding awkwardness	79	46	42	167
	Group identification, expressing personality	28	28	8	64
	Indicating, mocking, or self-mocking	38	27	4	69
	Keeping up with trends, as everyone is using them	30	25	12	67
	Starting or ending conversations	64	39	27	130
What are the sources of your emoji symbols?	System-provided emojis	81	83	84	248
	Downloaded from emoji stores	70	36	13	119
	Shared by friends	88	61	42	191
	Homemade chat emojis	28	28	0	56
	Downloaded from the internet	61	44	7	112

#### 4.1.2 Target audience and context of emoji symbol usage

People of all ages primarily use emojis with family, friends, and classmates, and less often with leaders or teachers. The choice of whom to use emojis with is influenced by age, identity, and social class, and depends on the closeness between the sender and receiver. Emojis are most frequently used in daily life to express emojis, preferences, and experiences. The main contexts for using emojis are when feeling relaxed and happy. Younger age (18–44 years old) use emojis more frequently in any situation. The type of emojis chosen varies with the context. Participants tend to use emojis to enhance emotions when feeling relaxed and happy, while their usage decreases when feeling angry or embarrassed. This indicates that emojis conveying positive emotions are more popular, while those for negative emotions and formal contexts are used less frequently.

#### 4.1.3 Motivations and sources of emoji symbol usage

Across generations, younger age groups find emoji usage lively and fun, enhancing communication atmosphere, and also use emojis to soften tone and avoid awkwardness. Middle-aged and elderly individuals find emojis convenient and quick, saving typing time. The data highlights three main reasons for using emojis: practicality, convenience, and entertainment. In terms of emoji sources, younger age groups mainly get emojis from friends and built-in systems, while middle-aged and elderly individuals mostly use built-in emojis. Overall, emoji usage frequency and purposes vary among different age groups, reflecting their social communication habits and preferences.

### 4.2 Comprehension bias in emojis

**Figure 1:**
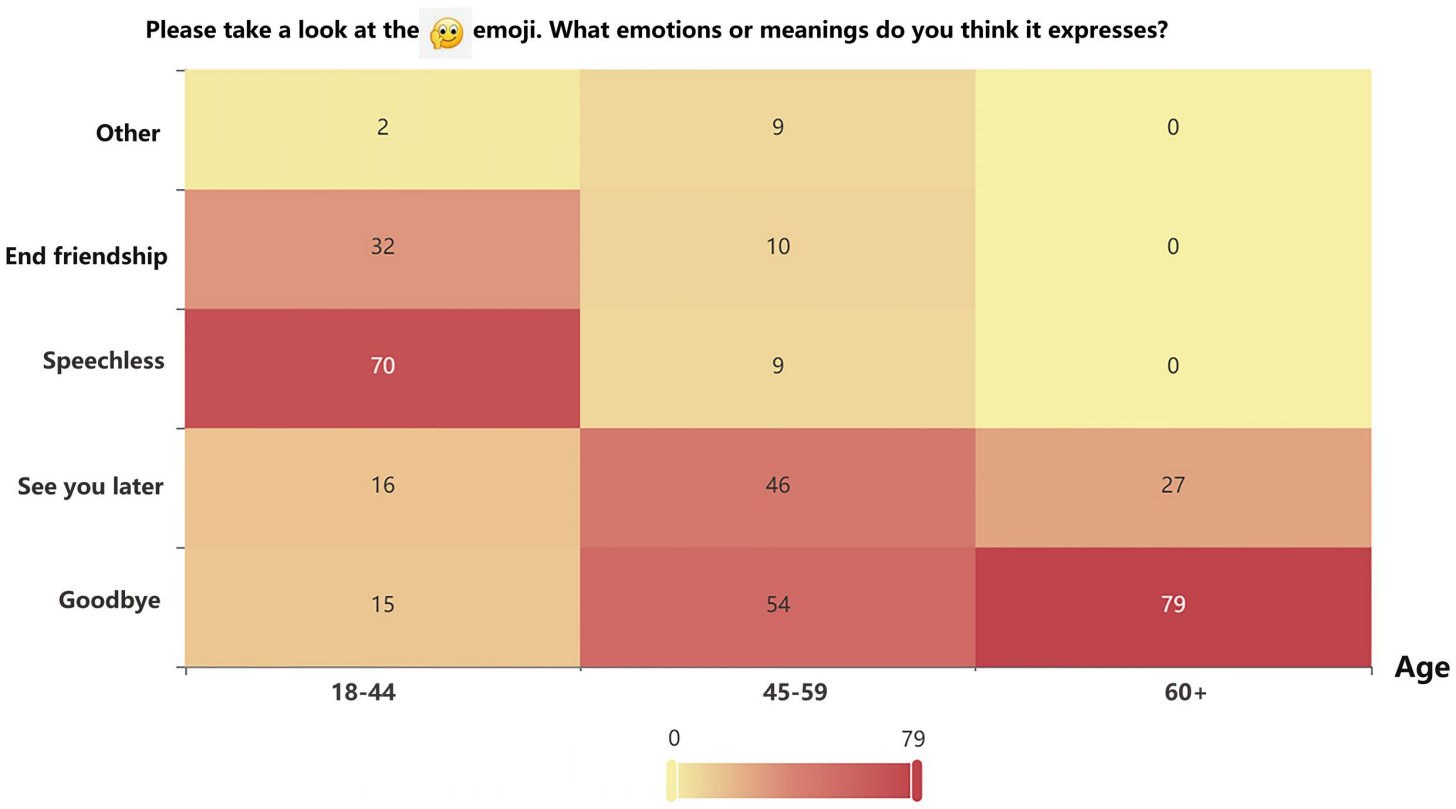
Heatmap analysis of the emotional significance of 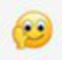 emoji.

We assessed participants’ emotional understanding of 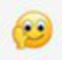 emoticons across different age groups. Based on *χ*^2^ test, the results of the expression of emotions or meanings based on 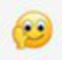 emojis show a significant effect (*p* < 0.001). The following figure displays a heatmap illustrating the emotional significance of 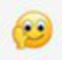 emoticons across generations, derived from values in a cross-contingency table. Regarding the emotional nuances conveyed through emojis 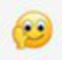, middle-aged and elderly individuals often perceive them as “goodbye” or “see you later.” Conversely, younger individuals frequently interpret emojis as signaling “speechlessness,” or even “end friendship,” introducing contrasting emotional layers. Middle-aged and elderly tend to interpret emoji symbols as simple emotional expressions, reflecting their familiarity with traditional emotional symbols. While younger people lean towards more complex emotions and social intentions, showcasing their open and diverse social identities, as shown in Figure 1. For emojis like 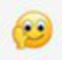 or 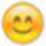 which can easily cause misunderstandings, unless middle-aged are already aware of their hidden meanings, the following phenomenon often occurs in conversations: parents send emojis that perplex their children, and parents do not understand the emojis their children send (Cavalheiro et al., 2023). This shows that a lack of common understanding between the sender and receiver leads to significant misinterpretations.

**Figure 2:**
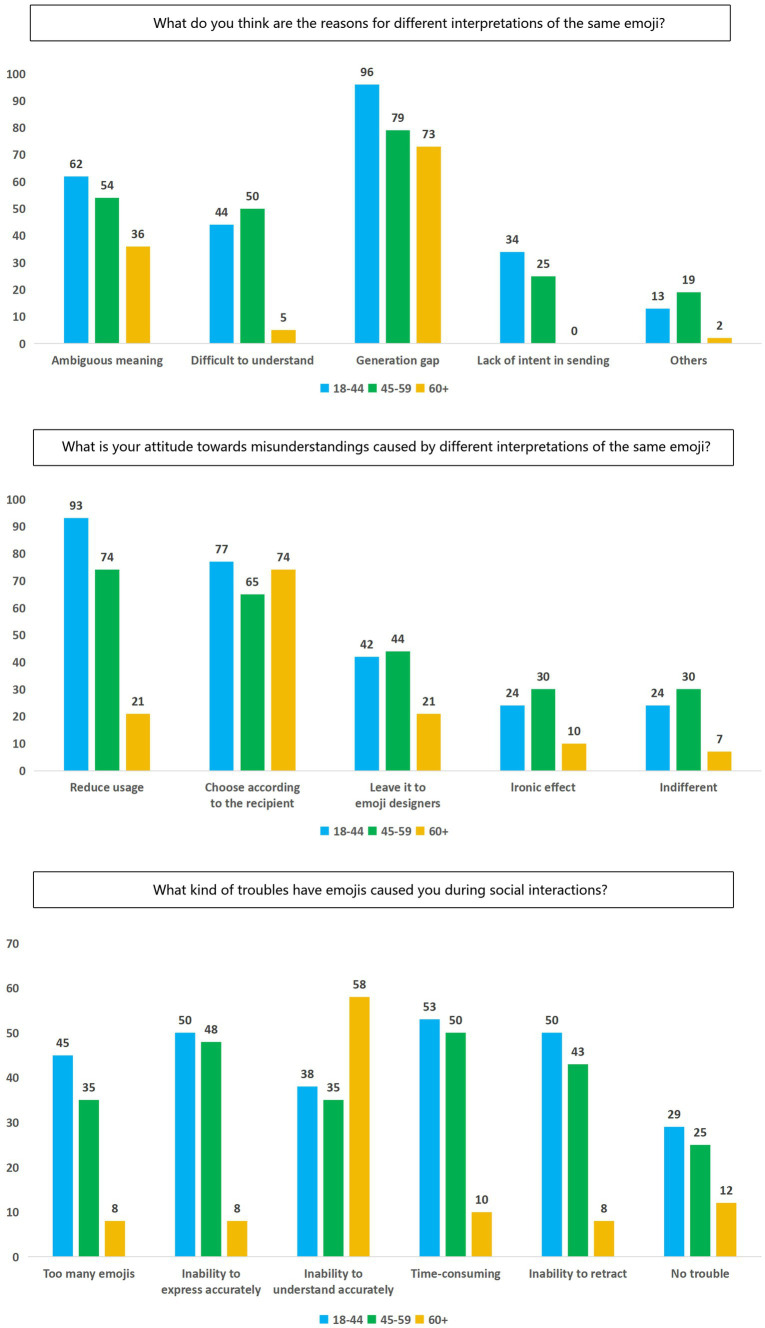
The issue of intergenerational understanding bias in emoji usage.

Statistical data indicates all age groups generally believe that generational gaps, cultural backgrounds lead to differing interpretations of emojis. Specifically, 96% of younger age groups, 79% of middle-aged, and 73% of elderly groups hold this view. The second most cited reason is the inherent ambiguity of emojis, with 62% of younger age groups, 54% of middle-aged groups, and 36% of elderly groups agreeing. Regarding misunderstandings caused by the same emoji, young and middle-aged groups prefer to avoid using ambiguous emojis (93% of younger age groups; 74% of middle-aged groups) and interpret meanings based on the recipient and context (77% of younger age groups; 65% of middle-aged groups). Elderly groups predominantly believe in interpreting meaning based on the recipient and context (74%). Different age groups face significant challenges with emoji usage, such as emojis being sent too frequently, causing missed important content. “unable to understand the meaning of others,” “spending a lot of time choosing appropriate emojis,” and “accidentally sending emojis that cannot be retracted.” Cultural backgrounds, social identity, communicative adaptation, and the inherent characteristics of emojis and users’ attitudes all influence how emojis are used and understood in social interactions, as shown in Figure 2.

Accurate communication necessitates a shared understanding between sender and receiver, termed the “common understanding space.” This implies that both parties possess a mutual comprehension of the symbolic meaning; otherwise, the communication process may falter, or comprehension biases may arise. To convey emojis without bias, both sender and receiver must hold consistent or analogous interpretations of the textual meaning conveyed by the same emoji. Consequently, the recipient can accurately apprehend the intended meaning behind the sender’s emoji. However, in numerous instances, due to the inherent ambiguity of the symbol itself and cognitive disparities between sender and receiver in terms of age and culture, recipients frequently grapple with correctly deciphering the sender’s intentions and attitudes initially. Cognitive disparities denote varying interpretations of the symbolic information transmitted between sender and receiver, often culminating in contradictory or conflicting readings (Cavalheiro et al., 2023).

### 4.3 Aesthetic preference in emojis

Aesthetic preferences are emotional, intellectual, and sensory all at once (Palmer et al., 2013). Although aesthetic preferences are important in human perception and play a crucial role in daily social decisions, the impact of the observer’s age on the aesthetic preferences for emojis has not been adequately studied. Based on*χ*^2^ test results, there are significant intergenerational differences in the collection, use, and preference of emoji types. In terms of emoji style collection, younger age significantly prefers “ambiguous, suitable for multiple contexts,” and this preference decreases significantly with age (*χ*^2^ = 52.776, *p* < 0.001). Users of all age groups show a significant preference for “clear graphic design, tailored to specific contexts,” but users aged 60+ are more inclined towards this type than users aged 18–44 (*χ*^2^ = 13.61, *p* < 0.001). “Novel, fun, lively” emojis are popular across all age groups, but younger users have a significantly higher preference for them compared to older users (*χ*^2^ = 13.171, *p* < 0.001). “Humorous, self-deprecating” emojis are more popular among younger users (*χ*^2^ = 22.242, *p* < 0.001). “reflect current trends and aesthetics” emojis are significantly more popular among younger users and decrease significantly with age (*χ*^2^ = 38.622, *p* < 0.001). In terms of frequently used emojis, classic yellow face emojis are used frequently across all age groups, with the highest usage among users aged 60+ (*χ*^2^ = 25.231, *p* < 0.001). Younger age use image-based emojis more frequently than middle-aged and older adults regarding preferred emoji themes, people aged 60+ prefer scenery (*χ*^2^ = 142.987, *p* < 0.001), while people aged 18–44 prefer cute pets (*χ*^2^ = 10.466, *p* < 0.005) and cartoon characters (*χ*^2^ = 15.168, *p* < 0.001). When expressing happiness, anger, or embarrassment middle-aged and older adults prefer classic yellow face emojis, while younger age prefer exaggerated, humorous, and absurd styles like collages, comic styles, film, and TV emojis. This indicates that younger age groups use a wider variety of emoji themes, as shown in Table 4.

**Table 4:** Analysis of intergenerational differences in aesthetic preferences for emojis.

	Age			Total	*χ* ^2^	*p*
	18–44	45–59	60+			
What style of emoji symbols would you collect?						
Ambiguous, suitable for multiple contexts	60 (44.44%)	29 (22.66%)	4 (3.77%)	93	52.776	<0.001
Clear graphic design, tailored to specific contexts	79 (58.52%)	69 (53.91%)	81 (76.42%)	229	13.61	0.001***
Novel, fun, lively	87 (64.44%)	64 (50.00%)	44 (41.51%)	195	13.171	0.001***
Humorous, self-deprecating	87 (64.44%)	68 (53.13%)	36 (33.96%)	191	22.242	<0.001
Reflect current trends and aesthetics	51 (37.78%)	45 (35.16)	5 (4.72%)	101	38.622	<0.001
What type of emoji symbols do you use most frequently?						
ASCII emojis	18 (13.33%)	18 (14.06%)	7 (6.6%)	43	3.717	0.156
(o(^@^)o)	10 (7.41%)	21 (16.41%)	5 (4.72%)	36	10.333	0.006***
Classic yellow face	62 (45.93%)	61 (47.66%)	80 (75.47%)	203	25.231	<0.001
Image-based emojis	54 (40.00%)	28 (21.88%)	42 (39.62%)	124	12.088	0.002***
Homemade emojis	24 (17.7%)	14 (10.94%)	6 (5.66%)	44	8.483	0.014**
Others	14 (10.37%)	12 (9.38%)	3 (2.83%)	29	5.284	0.071*
What themes of emoji symbols do you like?						
Natural landscapes	10 (7.41%)	59 (46.09%)	89 (83.96%)	158	142.987	<0.001
Flora and fauna	75 (55.56%)	47 (36.72%)	43 (40.57%)	165	10.466	0.005***
Cartoon characters	61 (45.19%)	40 (31.25%)	23 (21.70%)	124	15.168	0.001***
Internet celebrities	31 (22.96%)	5 (3.91%)	6 (5.66%)	42	28.483	<0.001
Absurdity	38 (28.15%)	5 (3.91%)	5 (4.72%)	48	43.156	<0.001
Others	15 (11.11%)	17 (13.28%)	7 (6.60%)	39	2.801	0.264

	Age			Total
	18–44	45–59	60+	
Which emoji symbol would you prefer to express happiness?				
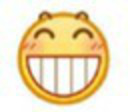	45	83	90	218
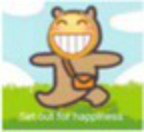	48	39	14	92
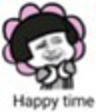	28	10	2	40
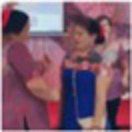	14	5	0	19
Which emoji symbol would you prefer to express anger?				
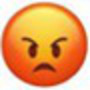	45	95	89	229
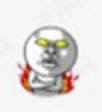	23	13	0	36
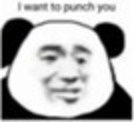	32	12	7	51
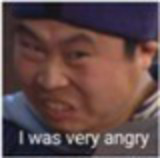	35	8	10	53
Which emoji symbol would you prefer to express awkwardness?				
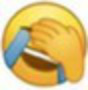	70	99	92	261
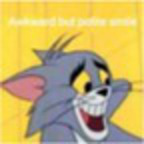	34	13	9	56
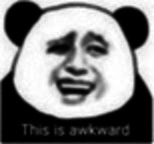	15	8	5	28
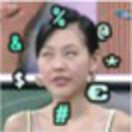	16	8	0	24
Have you used/liked similar emoji symbols before?				
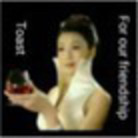	19	26	58	103
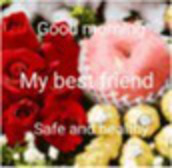	21	52	69	142
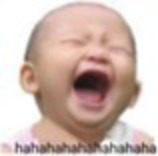	38	43	40	121
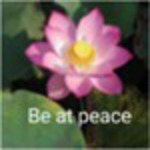	19	28	59	106
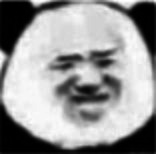	44	9	0	53
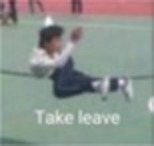	35	4	0	39
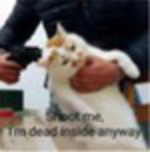	47	8	1	56
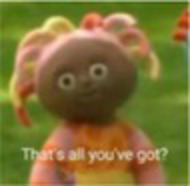	35	7	0	42

***, **, * represent significance levels of 1, 5, and 10%.

### 4.4 Emoji design preferences

We used the *χ*^2^ test to evaluate intergenerational preferences for different types and styles of emojis, as well as their design preferences and interpreted the results based on significance levels (*p*-value). In terms of participatory emoji design, there were no significant differences among age groups regarding the “combination of text and images, direct narrative design approach” (*χ*^2^ = 1.825, *p* < 0.402) Users aged 18–44 showed a significantly higher preference for “humorous entertainment and lively atmosphere” (*χ*^2^ = 13.571, *p* < 0.001). There were significant differences among age groups regarding the “unique style, showcasing personality design approach,” with users aged 18–44 and 60+ showing higher preferences than those aged 45–59 (*χ*^2^ = 7.599, *p* < 0.022). Users aged 60+ showed lower acceptance for “collage and deconstruction” and “referencing reality mimicking expressions” design styles (*χ*^2^ = 35.39, *p* < 0.001; *χ*^2^ = 32.469, *p* < 0.001). In terms of emoji colors, both young and middle-aged/older users preferred bright and lively colors. Additionally, young users (18–44 years) showed a significantly higher preference for “Color mixing, showcasing personality” compared to other age groups (*χ*^2^ = 22.692, *p* < 0.001). Regarding emoji fonts, younger age groups preferred artistic fonts and were more open to any font (*χ*^2^ = 5.447, *p* < 0.066; *χ*^2^ = 8.354, *p* < 0.015), while users aged 45–59 and 60+ preferred regular fonts like SimSun and SimHei (*χ*^2^ = 9.686, *p* < 0.008). The preferences for emoji designs among different age groups reflect their personalized needs and aesthetic tendencies, as shown in Table 5.

**Table 5:** Analysis of intergenerational emoji design preferences.

	Age			Total	*χ* ^2^	*p*
	18–44	45–59	60+			
If you were to participate in designing emoji, which design approach would you refer to?						
Text and graphics combination, direct narrative	60 (44.44%)	62 (48.44%)	42 (39.62%)	164	1.825	0.402
Humorous entertainment, lively atmosphere	91 (67.41%)	59 (46.09%)	53 (50.00%)	203	13.571	0.001***
Unique style, showcase personality	69 (51.11%)	52 (40.63%)	62 (58.49%)	183	7.599	0.022**
Collage and deconstruction, versatile elements	39 (28.89%)	24 (18.75%)	0 (0.00%)	63	35.39	<0.001
Real-life reference, emotion simulation	48 (35.56%)	52 (40.63%)	9 (8.49%)	109	32.469	<0.001
How do you prefer the color combination of emoji?						
Bright and lively colors	104 (77.04%)	91 (71.09%)	86 (81.13%)	281	3.309	0.191
Black and white contrast	37 (27.41%)	41 (32.03%)	16 (15.09%)	94	9.18	0.010**
Gray tones as primary, neutral wide range	33 (24.44%)	39 (30.47%)	0 (0.00%)	72	37.572	<0.001
Color mixing, showcasing personality	59 (43.70%)	51 (39.84%)	17 (16.04%)	127	22.692	<0.001
How do you prefer the font combination of emoji?						
Standard fonts like SimSun and SimHei	47 (34.81%)	43 (33.59%)	19 (17.92%)	109	9.686	0.008***
Artistic fonts, fancy Styles	48 (35.56%)	58 (45.31%)	53 (50.00%)	159	5.447	0.066*
Dynamic special effects fonts	36 (26.67%)	50 (39.06%)	31 (29.25%)	120	3.826	0.148
Any font is acceptable	52 (38.52%)	32 (25.00%)	44 (41.51%)	128	8.354	0.015**
No need for text, prefer pure emoji	37 (27.41%)	32 (25.00%)	18 (16.98%)	87	3.803	0.149
Other	9 (6.67%)	9 (7.03%)	0 (0.00%)	18	7.646	0.022**

***, **, * represent significance levels of 1, 5, and 10%.

Based on the above survey and analysis results, the design preferences of younger age groups can be summarized as follows: (1) fun elements: the primary motivation for younger age to use emojis is to increase social fun, enliven the social atmosphere, and convey social emotions. Therefore, whether an emoji is fun is the main consideration for this group. (2) Impactful design: younger age groups pursue individuality and unique styles. They prefer emojis with visually impactful, strong contrasts, dynamic exaggerations, bold colors, and those that integrate with internet pop culture. In addition to visual impact, they also favor the combination of text and images that create content conflict. (3) Everyday content: emojis often relate to everyday life topics. Emojis that depict study, work situations, daily life scenes, and other common aspects of ordinary life are more likely to gain emotional resonance with the audience.

Middle-aged and elderly individuals tend to use a fixed set of emoticon types that are less influenced by factors like online culture or personal identity. Internet trends and cultural shifts do not significantly impact emoticon updates among this demographic. In terms of design preferences, middle-aged and elderly individuals favor highly saturated and bright colors. Their emoticon choices often include landscapes, cute pets, cartoons, and anime, paired with artistic fonts or dynamic special effects fonts. Culturally, their narratives encompass polite greetings, ideological expressions, friendly blessings, tea and wine culture, and traditional festivals. They also prefer text forms that convey friendliness, such as morning and evening greetings or blessings. The emoticon usage patterns of Middle-aged groups closely align with their traditional lifestyle habits, including morning greetings, evening farewells, and inquiries like “Have you eaten?” when meeting acquaintances or old friends. Therefore, emoticons oriented towards daily life are particularly popular among middle-aged individuals, meeting both their social and practical needs.

## 5 Discussion

### 5.1 Comparison of emoji usage differences across different language backgrounds

Our research confirms the presence of comprehension and aesthetic biases across generations in emoji usage, supporting Hypothesis 1. Neel et al. (2023) indicate that emojis have a cognitive impact on emotionally neutral responses among American English, British English, and highly proficient non-native users of English. Barbieri et al. (2016) found that the meanings of the 150 most popular emojis are largely preserved across American English, British English, Spanish, and Italian, suggesting that emoji interpretation may be consistent across cultures. However, they noted slight differences in emoji interpretation between American and British English users. For example, British English users interpreted the wrapped gift emoji as a Christmas present, similar to other Christmas-related emojis like Santa Claus and Christmas tree, while American English users generally did not. Garcia et al. (2021) studied 96 native English speakers to explore young and older adults’ understanding of sarcasm in emojis. The results showed that both young and older adults understood and perceived sarcasm better when comments include emojis compared to when they did not. However, older adults had more difficulty correctly understanding sarcastic comments and their intent. Rodrigues et al. (2018) studied 505 Portuguese individuals and found that women were more likely to use emojis than men. Liu et al. (2020) studied native Chinese speakers, summarizing 10 types of dialogue situations where emojis were wrongly sent and 12 emotional components related to embarrassment. The results showed that (1) among the emotional components of embarrassment, shame has the highest explanation degree for embarrassment. (2) Males are more likely to be affected by embarrassment than females. (3) Users aged 18–25 and 26–30 years are more likely to be affected by embarrassment than those aged between 31 and 40 when they mistakenly send WeChat emojis. Our results are presented in the context of Gallud et al. (2018) findings, but aligning with those of Hsiao and Hsieh (2014), Barbieri et al. (2016), An et al. (2018), Liu et al. (2020), Garcia et al. (2021), and Neel et al. (2023). Our research extends the findings of previous studies by emphasizing generational differences in emoji interpretation and usage within a single cultural context. While prior studies focus on cross-cultural and gender differences, this study highlights the importance of understanding generational gaps to improve communication accuracy and emotional expression through emojis.

### 5.2 The relationship between social identity theory and intergenerational differences in emoji usage

SIT provides a framework for understanding how individuals establish their identities through interactions with specific social groups. Significant differences in emoji comprehension biases and aesthetic preferences between generations can be explained through SIT. These differences are mainly reflected in identity and belonging expression, intergroup comparison and differentiation, and expression of emotions and communication.

Identity and belonging expression: Individuals tend to use symbols and signs to express their sense of identity and belonging (Félonneau et al., 2013). In social media and online communication, younger generations use emojis more frequently to express complex emotions and social intentions, showcasing their unique group identity and sense of belonging. They prefer to use novel, interesting, and diverse emojis, reflecting not only their sensitivity to trends and technology, but also their need for belonging and recognition in social networks. In contrast, older generations tend to use traditional and familiar emojis, such as the classic yellow smiley face. This choice reflects their preference for stable and clear communication, and their use of these familiar symbols to express their social identity and maintain a sense of belonging. The use of emojis by older generations is more conservative, relying more on intuitive and straight forward symbols to ensure communication efficiency and accuracy.

Intergroup comparison and differentiation: SIT underscores comparisons between individuals and other groups to bolster intra-group identity (Jetten et al., 2004). Emoji choices reflect identification or comparison with different groups. Certain emojis may garner greater identity recognition due to cultural or group associations. The ambiguous nature of emojis and cognitive disparities across age and cultural backgrounds often complicate initial interpretation, affecting an accurate understanding of the sender’s intentions. These cognitive variations lead to divergent interpretations, underscoring social cognitive theory’s role in explaining emoticon use discrepancies among age groups. Younger generations compare themselves with older age groups through innovative and varied emoji usage, asserting uniqueness and superiority. Their use of diverse symbols showcases group creativity and openness to new trends, solidifying their social status. Conversely, older generations emphasize traditional values and clarity in communication, opting for familiar emojis. This reflects age-specific communication preferences and strengthens their group identity vis-a-vis other age cohorts.

Expression of emotions and communication: Emojis constitute a non-verbal symbol system (Tandyonomanu and Tsuroyya, 2018) capable of conveying emotions, attitudes, and tones in textual communication. This mode of expression not only enhances emotional nuances in communication but also transmits social signals and identity between groups. The younger generation employs emojis to convey a diverse range of emotions and social intentions, facilitating complex emotional exchanges and humorous expressions. This multifaceted use of symbols reflects their communication habits and social needs in the digital age. In contrast, the older generation uses emojis more for conveying simple and direct emotions, such as using a “smile” to express happiness or “goodbye” to bid farewell. This usage underscores clarity and directness in communication, aligning with their preference for efficiency and precision in communication.

The SIT highlights how an individual’s emoticon preferences are shaped by their affiliation with age-defined social groups, elucidating differences in intergenerational emoji usage and aesthetic preferences. Emojis serve as tools for expression, creativity, and social communication for the younger generation, while the older generation prioritizes clarity and efficiency in their use. Variances in emoji comprehension and aesthetic preferences between young and middle-aged individuals stem not only from personal choices but also from intricate interactions involving broader social contexts, cultural norms, generational identities, and social cognition (Zárate et al., 2019). Understanding these distinctions facilitates bridging communication divides and fostering improved intergenerational understanding in an increasingly digital world.

## 6 Conclusion

Intergenerational differences in emoji usage present communication barriers among individuals of varying age groups in interpersonal interactions. This study examines WeChat emojis, exploring comprehension and aesthetic differences between young and elderly users through questionnaire surveys. The findings reveal significant disparities in emoji usage habits, interpretation, and aesthetic preferences across generations. (1) Age differences influence the frequency and context selection of emoji usage. (2) Regarding interpretive differences, for instance, with the “goodbye” emoji, older adults translate it directly as “goodbye” or “see you later,” whereas most younger age groups understand it as “speechlessness” or “ending a friendship.” Middle-aged and elderly individuals tend to interpret goodbye emoji symbols as simple emotional expressions, while younger people lean towards complex emotions and social intentions. (3) In terms of aesthetic preferences, younger age use emojis frequently and have a wide variety, preferring humorous and exaggerated emojis. Middle-aged use emojis less frequently with a limited range, favoring traditional yellow smiley faces, natural scenery, and pet themes. Younger individuals lean towards aesthetics that are playful, conflict-oriented, and narrative-driven, whereas older adults prefer vibrant colors and emojis depicting everyday greetings. The differences in emoji comprehension and aesthetic preferences between young, middle-aged and, elderly individuals stem from the combined influences of social background, cultural norms, intergenerational identities, and social cognition. These findings advocate for real-time communication application developers to leverage these insights fully. They can do so by crafting suitable emojis, creating emojis devoid of biased interpretations, establishing a mutual cognitive realm between generations, and refining the accuracy and practical significance of emojis in conveying information and emotions during social exchanges. This study fosters amicable social interactions within real-time communication platforms, thereby contributing to the sustainable evolution of online social networking, and unveiling underlying psychological and social mechanisms.

Acknowledging the study’s limitations, we offer the following recommendations. Firstly, future research should employ effective sampling techniques to gather more samples aligning with research requirements. Secondly, when delineating the multifaceted meanings of emojis, researchers should consider additional influencing factors, such as ethnicity, geographical ties, and cultural backgrounds. This holistic consideration will aid in the broader application of research findings. Thirdly, forthcoming studies should compare the disparities and correlations in the opposite meanings of emojis across various languages and cultural contexts. Moreover, analyzing variances in the comprehension of opposing emoji meanings among users of different instant messaging applications is essential.

## Data availability statement

The data that support the findings of this study are available from the corresponding author, upon reasonable request.

## Ethics statement

The studies involving humans were approved by Research Ethics Committee of the Northeast Normal University. The studies were conducted in accordance with the local legislation and institutional requirements. The participants provided their written informed consent to participate in this study.

## Author contributions

DW: Writing – original draft, Formal analysis, Conceptualization, Resources, Methodology, Visualization. XXZ: Writing – review & editing, Methodology, Visualization, Data curation, Formal analysis. XJZ: Project administration, Supervision, Funding acquisition, Writing – review & editing.

## Footnotes

^1^ https://www.wjx.cn/
